# Superconcentrated NaFSA–KFSA Aqueous Electrolytes for 2 V-Class Dual-Ion Batteries

**DOI:** 10.1021/acsami.2c04289

**Published:** 2022-05-10

**Authors:** Tomooki Hosaka, Ayumi Noda, Kei Kubota, Kento Chiguchi, Yuki Matsuda, Kazuhiko Ida, Satoshi Yasuno, Shinichi Komaba

**Affiliations:** † Department of Applied Chemistry, Tokyo University of Science, Shinjuku-ku, Tokyo 162-8601, Japan; ‡ Elements Strategy Initiative for Catalysts and Batteries (ESICB), Kyoto University, Nishikyo-ku, Kyoto 615-8245, Japan; § Technova Inc., Chiyoda-ku, Tokyo 100-0011, Japan; ∥ Japan Synchrotron Radiation Research Institute (JASRI), SPring-8, 1-1-1 Kouto, Sayo-cho, Sayo-gun, Hyogo 679-5198, Japan

**Keywords:** sodium-ion battery, potassium-ion battery, aqueous electrolyte, SEI, hard X-ray photoelectron spectroscopy

## Abstract

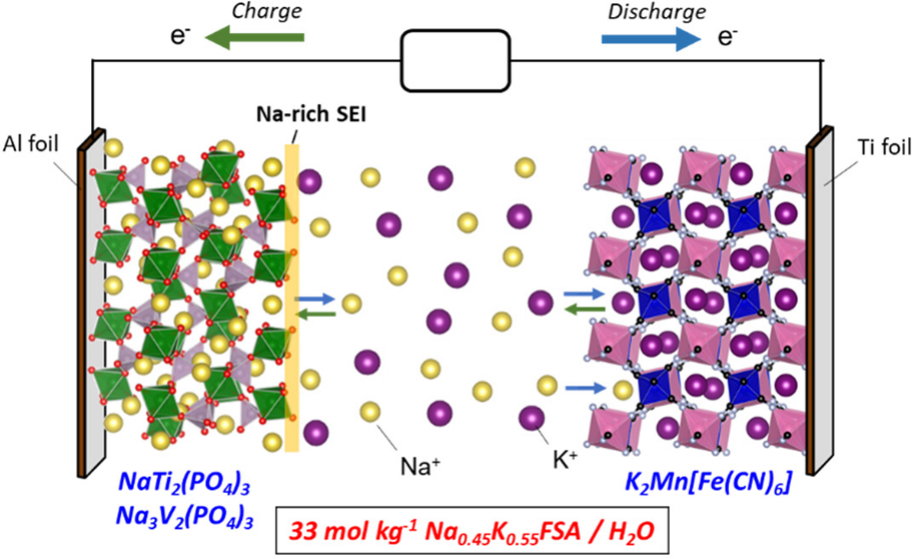

Superconcentrated aqueous electrolytes containing NaN(SO_2_F)_2_ and KN(SO_2_F)_2_ (for which sodium and potassium bis(fluorosulfonyl)amides (FSA), respectively, are abbreviated) have been developed for 2 V-class aqueous batteries. Based on the eutectic composition of the NaFSA–KFSA (56:44 mol/mol) binary system, the superconcentrated solutions of 35 mol kg^–1^ Na_0.55_K_0.45_FSA/H_2_O and 33 mol kg^–1^ Na_0.45_K_0.55_FSA/H_2_O are found to form at 25 °C. As both electrolytes demonstrate a wider potential window of ∼3.5 V compared to that of either saturated 20 mol kg^–1^ NaFSA or 31 mol kg^–1^ KFSA solution, we applied the 33 mol kg^–1^ Na_0.45_K_0.55_FSA/H_2_O to two different battery configurations, carbon-coated Na_2_Ti_2_(PO_4_)_3_∥K_2_Mn[Fe(CN)_6_] and carbon-coated Na_3_V_2_(PO_4_)_3_∥K_2_Mn[Fe(CN)_6_]. The former cell shows highly reversible charge/discharge curves with a mean discharge voltage of 1.4 V. Although the latter cell exhibits capacity degradation, it demonstrates 2 V-class operations. Analysis data of the two cells confirmed that Na^+^ ions were mainly inserted into the negative electrodes passivated by a Na-rich solid electrolyte interphase, and both Na^+^ and K^+^ ions were inserted into the positive electrode. Based upon the observation, we propose new sodium-/potassium-ion batteries using the superconcentrated NaFSA–KFSA aqueous electrolytes.

## Introduction

Aqueous Li-ion batteries using nonflammable aqueous electrolytes are promising for energy storage technology due to their intrinsic safety.^1,2^ Recently, superconcentrated aqueous electrolytes have attracted significant attention because of their wide potential window compared to that of conventional aqueous solutions. In 2015, Suo et al. reported that the superconcentrated aqueous electrolyte of 21 mol kg^–1^ lithium bis(trifuluoromethanesulfonyl) amide (N(SO_2_CF_3_)_2_^–^, TFSA^–^)/H_2_O demonstrated a wide potential window of 3.0 V, which realized a Mo_6_S_8_∥LiMn_2_O_4_ full cell with an operation voltage close to 2 V.^2^ Soon after that, Yamada et al. reported a binary anion system using TFSA^–^ and bis(perfluoroethysulfonyl)amide (N(SO_2_C_2_F_5_)_2_^–^, BETA^–^) anions to form a 27.8 mol kg^–1^ Li(TFSA)_0.7_(BETA)_0.3_ aqueous solution (Li(TFSA)_0.7_(BETA)_0.3_·2H_2_O), which demonstrated a Li_4_Ti_5_O_12_∥LiNi_0.5_Mn_1.5_O_4_ full cell with an operation voltage of ∼3.0 V.^3^ The wide potential window of superconcentrated aqueous solutions is achieved by both higher oxidation and reduction stability compared with the conventional concentration of electrolytes. The improved oxidation stability is attributed to the HOMO energy decrease caused by the coordination of water molecules to cations, whereas the reduction stability improvement would be due to the formation of anion-derived solid electrolyte interphase (SEI) induced by the higher LUMO energy of anions that have formed ionic pairs with cations.^2,3^

Similar to the Li electrolytes, superconcentrated aqueous Na and K electrolytes have been recently studied for battery application.^4−7^ In general, aqueous Na and K solutions show an ionic conductivity higher than that of Li counterparts because of their weak Lewis acidity, i.e., weak interaction between the cations and solvents/anions and consequent small Stokes radii of Na^+^ and K^+^ ions. Moreover, these cations with weak Lewis acidity allow the use of N(SO_2_F)_2_^–^ (FSA^–^) as a counteranion. The FSA^–^ anion has weak Lewis basicity, leading to high solubility and ionic conductivity, and forms a stable SEI derived from the decomposition products in nonaqueous Li, Na, and K batteries.^8−11^ However, the severe hydrolysis of FSA^–^ anions in the LiFSA aqueous solution to generate HF limits the utilization of LiFSA in aqueous electrolytes.^12^ In the concentrated NaFSA and KFSA electrolytes, the FSA^–^ anion exhibits higher stability against hydrolysis due to the weak interaction between the cation and the sulfonyl oxygen of FSA^–^.^7,12^ Although the long-term stability against hydrolysis still needs to be demonstrated, the FSA^–^ anion could be utilized for Na and K superconcentrated aqueous solutions. However, the demonstration of the 2 V-class aqueous Na or K batteries is still challenging owing to the narrower electrochemical stability windows of the electrolytes compared to those of the Li ones. The narrower electrochemical stability windows would be due to the following two reasons. First, the weaker interaction between the Na^+^/K^+^ ion and water molecules results in less HOMO energy and LUMO energy shift of the water molecules and anions, respectively.^13^ Second, there is a higher solubility of the corresponding fluorides, which are common SEI components, in Na and K aqueous solutions compared to that in Li solutions, with solubilities of LiF, NaF, and KF of 0.13, 4.13, and 102 g, respectively, in 100 mL of water.^14^ Recently, Zheng et al. reported Na and K superconcentrated aqueous solutions based on eutectic compositions of ternary anions, namely, TFSA^–^, (pentafluoroethanesulfonyl)(trifluoromethanesulfonyl)amide (N(SO_2_CF_3_)(SO_2_F_5_)^−^, PTFSA^–^), and trifluoromethanesulfonate (CF_3_SO_3_^–^, OTf^–^).^6^ The superconcentrated solutions of 18.5 mol kg^–1^ Na(PTFSA)_0.65_(TFSA)_0.14_(OTf)_0.21_ and 27.7 mol kg^–1^ K(PTFSA)_0.12_(TFSA)_0.08_(OTf)_0.8_ exhibited ionic conductivity of 14.0 and 34.6 mS cm^–1^, respectively.^6^ The conductivities are higher than that of 27.8 mol kg^–1^ Li(TFSA)_0.7_(BETA)_0.3_/H_2_O (3 mS cm^–1^).^3,6^ On the other hand, the potential window of the superconcentrated Na and K solutions was 2.7 and 2.5 V, which is still narrower than >3.0 V for the Li superconcentrated solutions.^2,3^

Not only anionic mixing but also cationic mixing decrease the melting point and increase the solubility^15−17^ because the larger entropy increase from the solid mixtures to the liquid mixtures at the eutectic mixture makes the liquid phase more stable.^18^ In dual-cation electrolytes such as Na–K systems, both cations could be inserted into active materials, realizing a wide variety of positive and negative electrode configurations. Moreover, the cation mixing should affect SEI stability due to the different solubility of the alkali metal compounds. Thus, it is important to clarify the effect of cation mixing on the solubility of the salts, potential window, reaction mechanism of active materials, and the SEI composition and stability. In this study, we prepared NaFSA–KFSA/H_2_O electrolytes based on the eutectic composition of the NaFSA–KFSA (56:44 mol/mol) binary system. A series of superconcentrated Na_*x*_K_1–*x*_FSA aqueous solutions were evaluated in terms of solubility, ionic transport properties, FSA^–^ stability against hydrolysis, electrochemical stability, and SEI formation to prove the impact of mixed Na^+^ and K^+^ ions. Through the use of superconcentrated Na_*x*_K_1–*x*_FSA solution, 2 V-class dual-cation batteries of carbon-coated NaTi_2_(PO_4_)_3_ (NTP-C)∥K_2_MnFe(CN)_6_] (KMnHCF) and carbon-coated Na_3_V_2_(PO_4_)_3_ (NVP-C)∥KMnHCF were demonstrated on the basis of understanding the electrode reaction including mobile ionic species and the surface layer of the electrode.

## Experimental Methods

### Electrolyte Preparation

A series of Na_1–*x*_K_*x*_FSA aqueous solutions were prepared by dissolving NaFSA (Solvionic) and KFSA (Solvionic) into deionized water at various concentrations at 25 ± 1 °C.

### Electrode Material Synthesis

The K_2_Mn[Fe(CN)_6_] positive electrode material was synthesized via a chelate-assisted precipitation method.^10,19^ Briefly, 4 mmol K_4_Fe(CN)_6_·3H_2_O and 4 mmol MnCl_2_·4H_2_O were separately dissolved in 100 mL of 0.2 M potassium citrate solutions. Then these two solutions were mixed by dropping the MnCl_2_ solution at 0.5 mL min^–1^ into the K_4_Fe(CN)_6_ solution with magnetic stirring under a N_2_ atmosphere at 25 ± 3 °C. After being stirred for 15 h, the precipitate was centrifuged, filtered, and washed thoroughly with 400 mL of deionized water. Finally, the final product was obtained after vacuum drying at 100 °C for 24 h.

NASICON-type materials of NaTi_2_(PO_4_)_3_ (NTP) and Na_3_V_2_(PO_4_)_3_ (NVP) were used for the negative electrode. The NTP was synthesized via a sol–gel method.^20,21^ Specifically, 0.01 M titanium butoxide (Aldrich) was dissolved in 40 mL of 30% H_2_O_2_ and 15 mL of 28% NH_3_ under constant agitation. Citric acid (FUJIFILM Wako Pure Chemical) was added to the solution at a molar ratio of citric acid to titanium ions of 2:1. After sufficient stirring, stoichiometric amounts of NH_4_H_2_PO_4_ (FUJIFILM Wako Pure Chemical) in 10 mL of distilled water and Na_2_CO_3_ (Nacalai Tesque) in 10 mL of distilled water were added. Then ethylene glycol (Kishida Chemical) was added to the mixture to give a molar ratio of 1:1 with the citric acid. The resulting mixture was kept at 80 °C for 2 h under continuous stirring. The obtained transparent gel was heated at 140 °C for 2 h. The gel precursor was subsequently decomposed at 350 °C for 3 h in air, which led to the elimination of the organic contents. The residual powders were ground and calcined at 800 °C for 12 h in air to obtain NaTi_2_(PO_4_)_3_ powders. To improve the electronic conduction of NTP by carbon coating, the synthesized NTP powders were dispersed in a 50% sucrose aqueous solution, and the solution was heated in a polytetrafluoroethylene (PTFE)-lined autoclave at 180 °C for 6 h. The precipitates were filtered, washed, and recalcined at 800 °C for 1 h in an Ar atmosphere to obtain carbon-coated NTP (NTP-C). The amount of carbon in the NTP-C was estimated to be 3% by thermogravimetric analysis using a thermogravimetric analyzer (DTG-60, Shimadzu).

The NVP-C composite was directly synthesized via the soft template method as reported before.^22^ Cationic surfactant of cetyltrimethylammonium bromide (CTAB, FUJIFILM Wako Pure Chemical) was dissolved in a mixture of deionized water and ethanol. To this solution was added a stoichiometric mixture of CH_3_COONa (Kanto Chemical), vanadium(III) acetylacetonate (VO(C_5_H_7_O_2_)_2_, Strem Chemicals), and NH_4_H_2_PO_4_ with continuous and vigorous stirring. The resulting precipitate was then continuously stirred for several hours and dried at 70 °C. The precipitate was calcined in an Ar atmosphere at 750 °C for 6 h. The carbon content in NVP-C was estimated to be 1.9% by thermogravimetric analysis.

### Electrochemical Measurement

The KMnHCF electrodes consisting of a mixture of 70 wt % KMnHCF, 20 wt % Ketjen black (KB, Carbon ECP, Lion), and 10 wt % poly(vinylidene fluoride) (PVDF, #9100, Kureha) were prepared with a coating mixture slurry on Ti foil and drying the coat at 150 °C under vacuum. The electrodes consisting of a mixture of 70 wt % NTP-C and NVP-C as active material, 25 wt % acetylene black (AB, Li-400, Denka), and 5 wt % PVDF were prepared by coating a mixture slurry on Al foil and drying the coat at 150 °C under vacuum. Activated carbon electrodes consisting of 80 wt % activated carbon (YP50F, Kuraray), 10 wt % KB, and 10 wt % PTFE (Daikin) were formed on Al-expanded metal and dried at 200 °C under vacuum. Voltammetry was conducted in the three-electrode cell (SB1A, EC FRONTIER) in which the activated carbon counter electrode and Ag/AgCl reference electrode were used. Charge–discharge tests were conducted in three-electrode cells (SB9, EC FRONTIER) with a Ag/AgCl reference electrode and glass fiber separator (GB-100R, Advantec).

### Surface, Morphology, and Composition Analysis

Hard X-ray photoelectron spectroscopy (HAXPES) spectra of the tested NTP-C electrodes were acquired by high excitation energy of hard X-ray, 7939 eV, and a photoelectron energy analyzer of R-4000 (Scienta Omicron) at BL46XU in SPring-8, Japan. The photoelectron detection angle and pass energy of the analyzer were set to 80° and 200 eV, respectively. Electrochemically tested NTP-C electrodes were taken out from cycled three-electrode cells and rinsed with propylene carbonate and dimethyl carbonate in a N_2_-filled glovebag to avoid air exposure. Then the electrodes were dried at room temperature under vacuum and transferred using a transfer vessel to avoid air exposure. The detailed setup and condition of the HAXPES measurement are described in our previous paper.^23,24^ The binding energy of the obtained spectrum was calibrated with the binding energy of sp^2^ carbon of graphite being 284.3 eV.

The structural change of KMnHCF electrodes after the electrochemical test was investigated using an X-ray diffractometer (Smart Lab, Rigaku) equipped with a high-speed one-dimensional X-ray detector D/teX Ultra. The chemical composition of the electrodes was characterized by energy-dispersive X-ray (EDX) spectroscopy using a scanning electron microscope (SEM, JCM-6000, JEOL). In these ex situ experiments, the KMnHCF electrodes were immediately taken out from the three-electrode cells within 3 min after reaching the target potentials without potential holding to avoid the relaxation and ionic exchange. Then the electrodes were rinsed with dimethyl sulfoxide and dimethyl carbonate in a N_2_-filled glovebag and dried under vacuum.

## Results and Discussion

**Figure 1:**
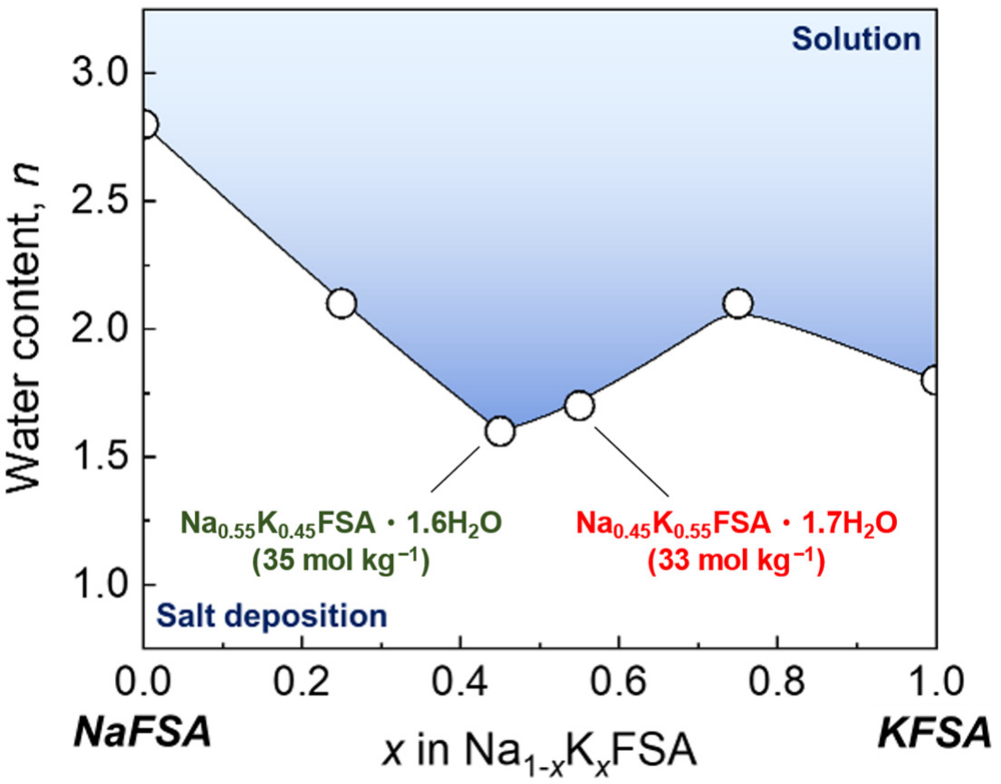
Liquid-salt deposition line of Na_1–*x*_K_*x*_FSA salt–water mixtures. The solubility is experimentally examined through the gradual addition of water and stirred for 12 h at 25 °C.

First, the solubility of the Na_1–*x*_K_*x*_FSA mixture in water was examined at different Na/K ratios. Figure 1 shows the water content of saturated aqueous Na_1–*x*_K_*x*_FSA solutions at 25 °C. The concentrations of the saturated NaFSA and KFSA solutions as end-members are 20 and 31 mol kg^–1^, respectively. By mixing Na^+^ and K^+^ cations, higher concentration solutions of 35 mol kg^–1^ Na_0.55_K_0.45_FSA/H_2_O and 33 mol kg^–1^ Na_0.45_K_0.55_FSA/H_2_O were demonstrated. The NaFSA/KFSA ratio for the highest concentration solution in the NaFSA–KFSA–H_2_O system is quite close to the eutectic composition of the binary NaFSA/KFSA = 56/44 in molar ratio,^25^ which should be mainly due to the largest entropy increase from the solid mixtures to the liquid mixtures, i.e., the molten salt or aqueous solution, at this NaFSA/KFSA ratio.^18^ On the other hand, Na_0.25_K_0.75_FSA shows solubility lower than that of the KFSA in H_2_O, which is different from the melting point behavior of the binary NaFSA–KFSA system. Similar behavior to the ternary system has been reported for the LiNO_3_–NaNO_3_–H_2_O^26^ and Mg(NO_3_)_2_–NaNO_3_–H_2_O^27^ systems, and the lower solubility of Na_0.25_K_0.75_FSA would be due to existence of a stable NaFSA hydrate phase.^28^Table 1 shows ionic conductivity and viscosity at 25 °C of the series of superconcentrated Na_1–*x*_K_*x*_FSA solutions. To compare the effect of cations on ionic conductivity, we also prepared 20 mol kg^–1^ solutions of NaFSA, Na_0.55_K_0.45_FSA, and KFSA. The 20 mol kg^–1^ KFSA solution showed the highest ionic conductivity of 75.3 mS cm^–1^, whereas the 20 mol kg^–1^ NaFSA solution showed the lowest ionic conductivity (41.3 mS cm^–1^). The high ionic conductivity of the K solution is due to the weak interaction of K^+^ ions with either water molecules or anions. The 20 mol kg^–1^ Na_0.55_K_0.45_FSA solution delivers ionic conductivity of 49.8 mS cm^–1^, which was lower than the ionic conductivity linearly predicted from the Na/K ratio (∼57 mS cm^–1^). The significant decrease in ionic conductivity from the KFSA solution to Na_0.55_K_0.45_FSA solution corresponds to an increase in viscosity, which may be due to ion-pair formation between Na^+^ and FSA^–^. Indeed, the viscosities of the 31 mol kg^–1^ KFSA solution and 20 mol kg^–1^ NaFSA were almost identical despite a much higher concentration of the KFSA solution. The 31 mol kg^–1^ KFSA solution delivered a relatively high ionic conductivity of 43.4 mS cm^–1^. The 35 mol kg^–1^ Na_0.55_K_0.45_FSA and 33 mol kg^–1^ Na_0.45_K_0.55_FSA solutions exhibited ionic conductivities of 22.0 and 25.2 mS cm^–1^, respectively. It is worth mentioning that the ionic conductivities are much higher than 3 mS cm^–1^ for 27.8 mol kg^–1^ Li(TFSA)_0.7_(BETA)_0.3_.^3^ The 33 mol kg^–1^ Na_0.45_K_0.55_FSA solution showed a viscosity of about 34 mPa·s, which was lower than the 47 mPa·s of the 35 mol kg^–1^ Na_0.55_K_0.45_FSA solution. The reason behind the low viscosity of the Na_0.45_K_0.55_FSA solution is the higher K ratio as well as the slightly lower concentration. Notably, these Na_1–*x*_K_*x*_FSA superconcentrated solutions showed viscosity far lower than that of the Li superconcentrated solution (203 mPa·s), despite the higher concentration for Na_1–*x*_K_*x*_FSA. Moreover, preliminary semiquantitative analysis of F^–^ ion in the electrolytes using the Zr-EDTA complex^29^ showed that the 33 mol kg^–1^ Na_0.45_K_0.55_FSA solution contains negligible F^–^ ion (∼3 ppm) after 2 weeks storage at 25 ± 3 °C (see Supporting Information, Figure S1). The F^–^ ion concentration is much lower than that of 31 mol kg^–1^ LiFSA solution (exceeding the upper limit of calibratable F^–^ concentration, >6 ppm). Therefore, it was proved that the utilization of Na^+^ and K^+^ ions, which have Lewis acidity weaker than that of Li^+^ ions, demonstrates superconcentrated electrolytes showing higher ionic conductivity, lower viscosity, and higher FSA^–^ stability.

**Table 1:** Molar Ratio, Ionic Conductivity, and Viscosity of the Prepared Na_1–*x*_K_*x*_FSA Solutions and a Reported Li Solution

	molar ratio (H_2_O/sum of cation)	ionic conductivity (mS cm^–1^)	viscosity (mPa·s)
20 mol kg^–1^ NaFSA	2.8	41.3	17.72
20 mol kg^–1^ Na_0.55_K_0.45_FSA	2.8	49.8	17.47
20 mol kg^–1^ KFSA	2.8	75.3	11.84
31 mol kg^–1^ KFSA	1.8	43.4	17.12
35 mol kg^–1^ Na_0.55_K_0.45_FSA	1.6	22.0	46.84
33 mol kg^–1^ Na_0.45_K_0.55_FSA	1.7	25.2	34.36
Li(TFSA)_0.7_(BETA)_0.3_^3^	2	3.0	203

**Figure 2:**
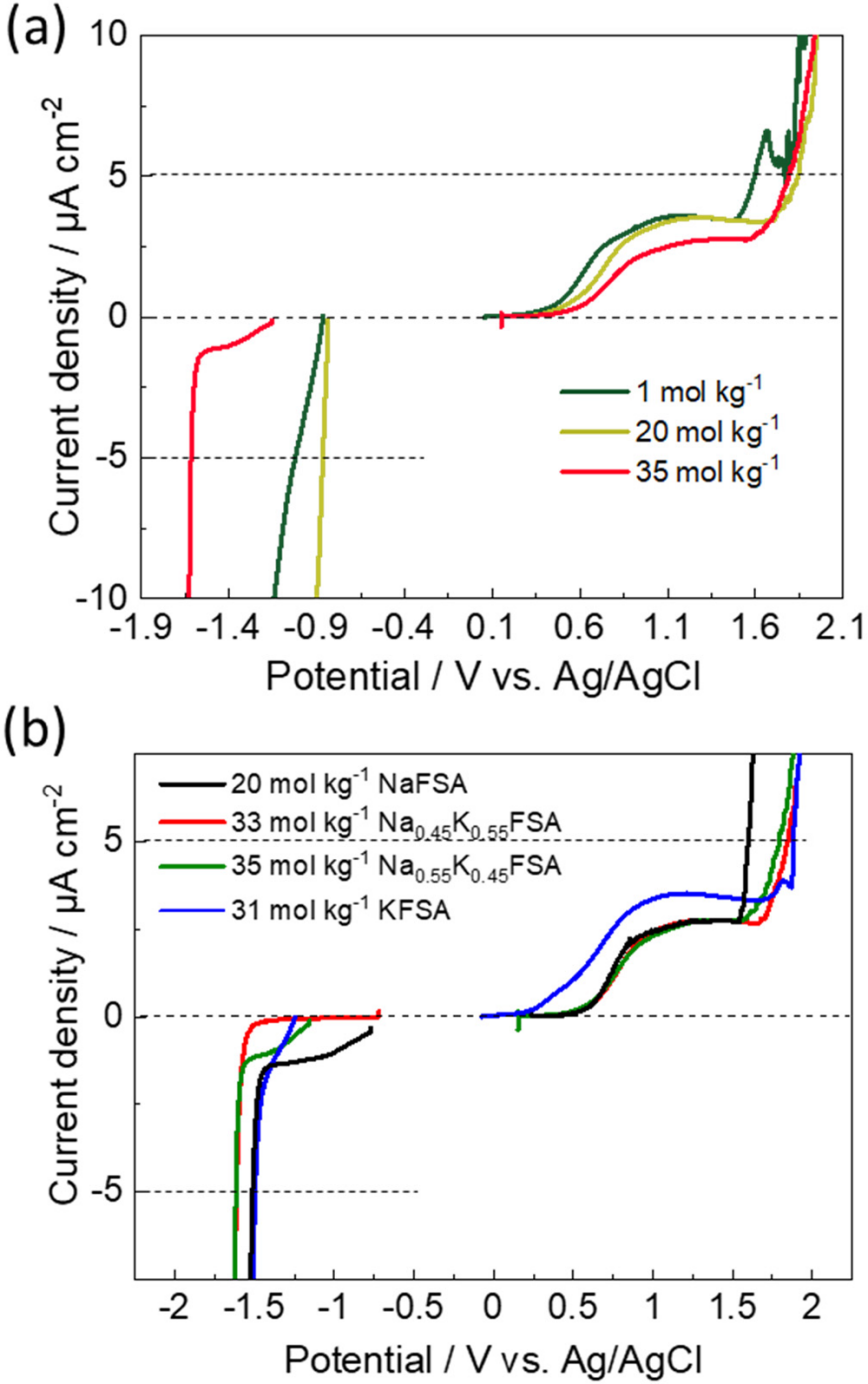
(a) LSV curves in Na_0.55_K_0.45_FSA/H_2_O solutions at different concentration. (b) LSV curves in the saturated Na_1–*x*_K_*x*_FSA solutions at a scan rate of 0.5 mV s^–1^. The working electrodes were Al and Ti foils for cathodic and anodic scan, respectively. The LSV measurements started from the open circuit potential.

To test their potential window, the anodic and cathodic stability of the electrolytes was evaluated by slow linear sweep voltammetry (LSV) measurements at a scan rate of 0.5 mV s^–1^ using the working electrodes of Ti and Al foils, respectively. First, 1, 20, and 35 mol kg^–1^ Na_0.55_K_0.45_ solutions were evaluated to check the concentration effect, as shown in Figure 2a. In the anodic scan, the 1 mol kg^–1^ solution exhibited a current increase from 1.5 V vs Ag/AgCl, and the current reached 5 μA cm^–2^ at 1.60 V vs Ag/AgCl. In this study, 5 μA cm^–2^ was used to simply define the oxidation and reduction stability limit potentials of the electrolytes. In contrast to the 1 mol kg^–1^ solution, the higher concentration solutions of 20 and 35 mol kg^–1^ exhibited oxidation stabilities of ∼1.8 V vs Ag/AgCl that were higher than those of the 1 mol kg^–1^ one (Figure 2a), and no clear difference was confirmed between the 20 and 35 mol kg^–1^ solutions. It is worth noting that a small and almost constant anodic current of 2–3 μA cm^–2^ was observed in all electrolytes at a potential range of around 0.5–1.4 V vs Ag/AgCl, indicating that side reactions such as passivation occurred on the Ti electrode. Indeed, the constant anodic current disappeared when a Pt electrode was used instead of a Ti electrode (Figure S2). In contrast to the anodic stability, significant differences during the cathodic sweep were found in reduction stability between 20 and 35 mol kg^–1^ electrolytes. The 1 and 20 mol kg^–1^ solutions showed lower stability against electroreduction, showing an increase in cathodic current below −1.0 V vs Ag/AgCl (Figure 2a). In contrast, the 35 mol kg^–1^ solution demonstrated significant improvement in reduction stability down to −1.62 V vs Ag/AgCl, indicating the passivation layer formation according to the previous observation.^3^

Figure 2b shows LSV curves of the saturated solutions. In the anodic scan, the 20 mol kg^–1^ NaFSA solution showed current flow from ∼1.5 V vs Ag/AgCl. The higher concentration solutions of 33 mol kg^–1^ Na_0.45_K_0.55_FSA, 35 mol kg^–1^ Na_0.55_K_0.45_FSA, and 31 mol kg^–1^ KFSA exhibited anodic stability higher than that of the NaFSA solution (Figure 2b). On the other hand, 35 mol kg^–1^ Na_0.55_K_0.45_FSA and 33 mol kg^–1^ Na_0.45_K_0.55_FSA solutions showed cathodic stability of −1.62 V vs Ag/AgCl, which was higher than that of 31 mol kg^–1^ KFSA and 20 mol kg^–1^ NaFSA, which implies that passivation layer is more stable in the cation–eutectic solutions than the end-members. Overall, the 33 mol kg^–1^ Na_0.45_K_0.55_FSA and 35 mol kg^–1^ Na_0.55_K_0.45_FSA solutions demonstrate the widest potential window of 3.5 V.

**Figure 3:**
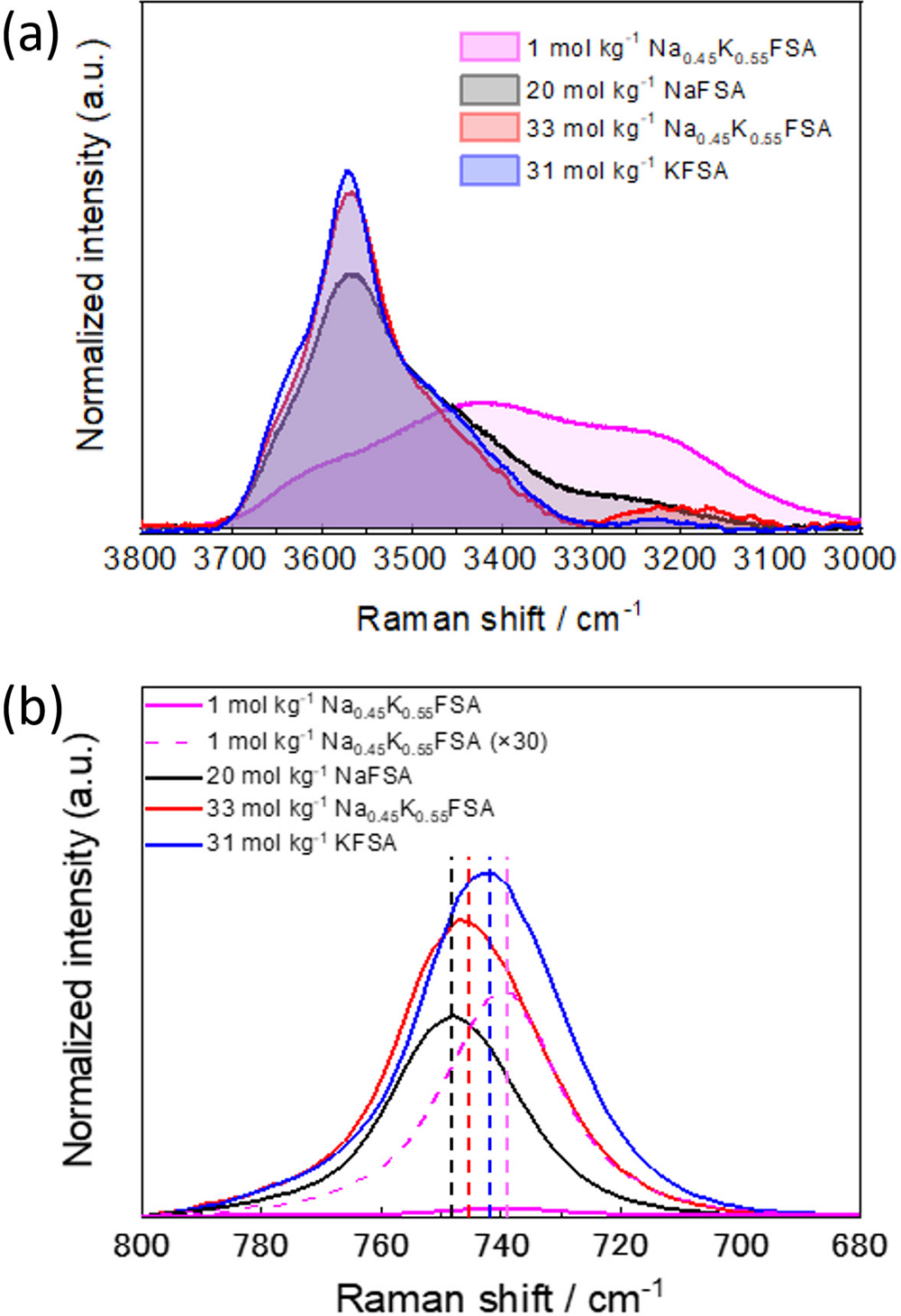
Raman spectra of 1 mol kg^–1^ Na_0.45_K_0.55_FSA, 20 mol kg^–1^ NaFSA, 33 mol kg^–1^ Na_0.45_K_0.55_FSA, and 31 mol kg^–1^ KFSA solutions in the range of (a) 3000–3800 cm^–1^ and (b) 680–800 cm^–1^.

To understand the different oxidation and reduction stability of the solutions, we investigated the solution structures of 20 mol kg^–1^ NaFSA, 31 mol kg^–1^ KFSA, and 33 mol kg^–1^ Na_0.45_K_0.55_FSA with Raman spectroscopy. Figure 3a shows Raman spectra attributed to the O–H stretching vibration of H_2_O, which reflects the coordination structure of water molecules. All Raman spectra were normalized by the integrated area in the range of 3000–3800 cm^–1^. The spectra of 1 mol kg^–1^ Na_0.45_K_0.55_FSA solution exhibited broad peaks in the range of 3000–3700 cm^–1^, which are typically observed for bulk water and attributed to several structures in a water cluster.^3,30^ The 20 mol kg^–1^ NaFSA, 31 mol kg^–1^ KFSA, and 33 mol kg^–1^ Na_0.45_K_0.55_FSA solutions exhibited a decrease in intensity around 3000–3300 cm^–1^ and an increase in the peak at 3570 cm^–1^, which should be attributed to smaller water clusters^31,32^ and is similar to crystalline hydrates.^30^ Thus, the sharp peak indicates that the water clusters were isolated into small clusters or monomers by coordinating to cations in the superconcentrated solutions, which would be the key for extended oxidation stability and suppression of SEI dissolution.^3,33^ Comparing the three superconcentrated solutions, 31 mol kg^–1^ KFSA and 33 mol kg^–1^ Na_0.45_K_0.55_FSA showed a pronounced sharp peak at 3570 cm^–1^ compared to that of 20 mol kg^–1^ NaFSA, indicating less free water molecules in the KFSA and Na_0.45_K_0.55_FSA. These solution structures reasonably explain the fact that 31 mol kg^–1^ KFSA and 33 mol kg^–1^ Na_0.45_K_0.55_FSA showed an oxidation stability higher than that of the 20 mol kg^–1^ NaFSA, as shown in Figure 2b.

In addition to the coordination structure of water molecules, that of FSA^–^ anions was evaluated. Figure 3b shows Raman spectra attributed to the S–N–S bending vibration of the FSA^–^ anion, which reflects the coordination structure of the FSA^–^ anions. The 1 mol kg^–1^ solution shows a single peak located at 738 cm^–1^, which can be attributed to less aggregated anions such as a solvent-separated ion pair (SSIP) and a contact ion pair (CIP).^34^ In the 20 mol kg^–1^ NaFSA, 31 mol kg^–1^ KFSA, and 33 mol kg^–1^ Na_0.45_K_0.55_FSA solutions, the peak exhibited an apparent blue shift (Figure 3b), suggesting significant ion-pair formations like the aggregate (AGG).^34,35^ The 20 mol kg^–1^ NaFSA and 33 mol kg^–1^ Na_0.45_K_0.55_FSA solutions showed an apparent blue shift larger than that of the 31 mol kg^–1^ KFSA solution, indicating a relatively strong interaction and significant ion-pair formation between Na^+^ and FSA^–^ ions. Previous studies proved that these ion pairs promote the reductive decomposition of anions to form the SEI layer.^3,6,33^ Thus, the FSA^–^-derived SEI formation would be successfully induced in 33 mol kg^–1^ Na_0.45_K_0.55_FSA compared with 31 mol kg^–1^ KFSA solution, resulting in successful passivation as well as higher reduction stability. Moreover, as the solubility of NaF is much lower than that of KF, the passivation layer in the dual-cation solutions would be more stable than in the KFSA solution, which will be further discussed later. Therefore, the lower solubility of NaF compared to that of KF and the solution structure with few free water molecules and the significant formation of AGGs would be the key factors that led to the wide potential window of Na_1–*x*_K_*x*_FSA solutions.

**Figure 4:**
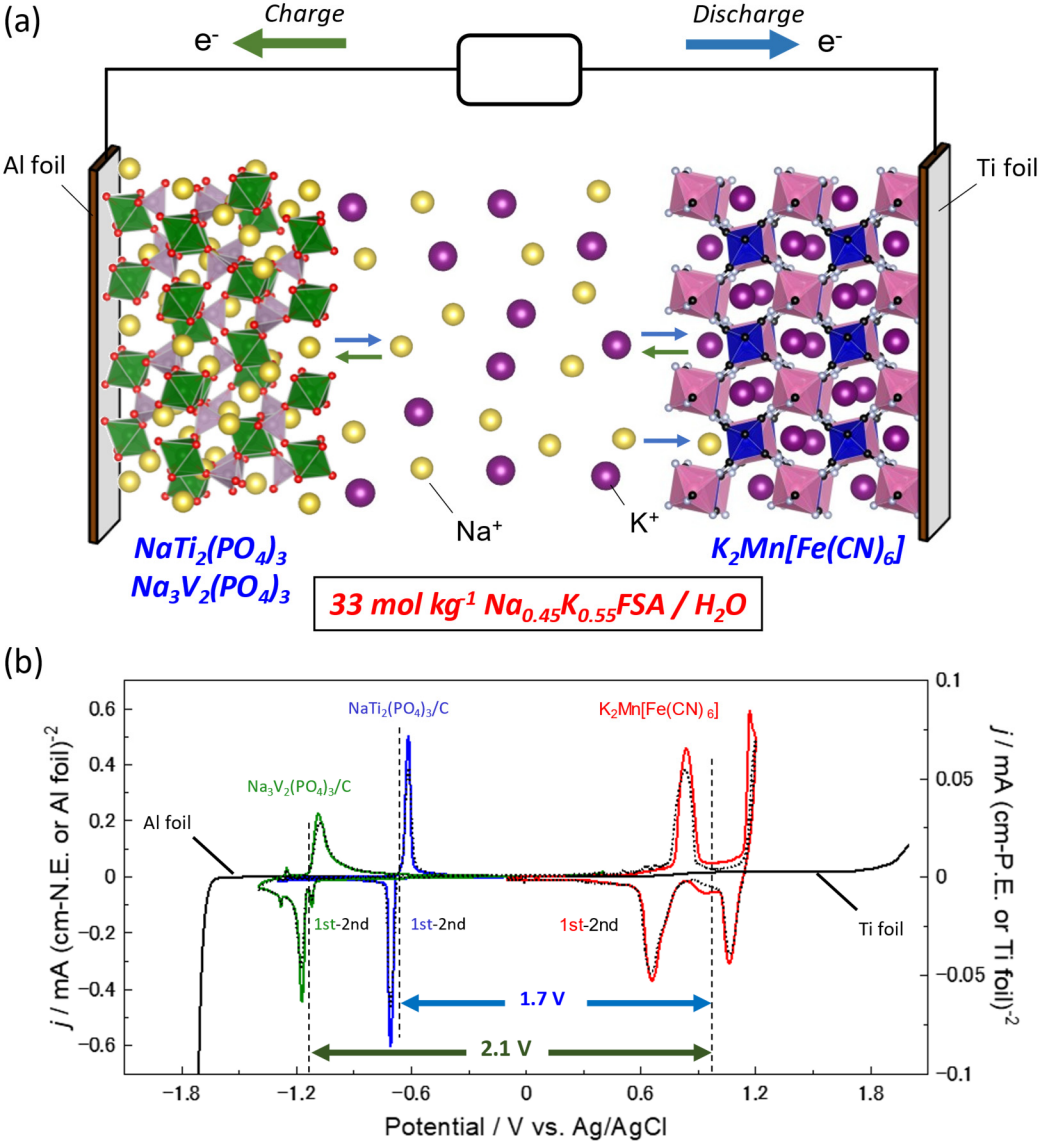
(a) Schematic illustration of aqueous Na/K dual-ion battery fabricated in this study. (b) Cyclic voltammograms of the positive (KMnHCF) and negative (NTP-C and NVP-C) electrodes in 33 mol kg^–1^ Na_0.45_K_0.55_FSA solution at a scan rate of 0.1 mV s^–1^. The LSV curves of Ti and Al foil for the respective anodic and cathodic scans are also shown.

As the 33 mol kg^–1^ Na_0.45_K_0.55_FSA and 35 mol kg^–1^ Na_0.55_K_0.45_FSA exhibited superior properties of the wide potential window and high ionic conductivity, we selected 33 mol kg^–1^ Na_0.45_K_0.55_FSA showing higher ionic conductivity for battery tests. The Na_0.45_K_0.55_FSA electrolyte is thought to be compatible with electroactive insertion materials of Na^+^, K^+^, H^+^, FSA^–^, and OH^–^ ions. Because we have studied sodium and potassium insertion materials in the past decade,^36,37^ we designed the Na/K dual-ion battery and evaluated the electrochemical reaction of the electrode materials, as schematically illustrated in Figure 4a. Taking the working potential and chemical stability against water into account, we selected KMnHCF as the positive electrode material, and the NASICON-type polyanionic compounds, NTP^21,38,39^ or NVP,^22,40^ were used as the negative electrode material. KMnHCF is known as a potassium and sodium insertable material,^41−43^ and NTP and NVP are sodium insertion materials.^21,22,38,40^Figure 4b shows the overall potential–current diagram of the electrode materials tested under cyclic voltammetry conditions in 33 mol kg^–1^ Na_0.45_K_0.55_FSA electrolyte in comparison with the LSV curves of Ti or Al foil electrodes used as current collectors. Three of these positive and negative electrode materials successfully showed a reversible redox behavior without any apparent irreversible reaction because the redox activity of the selected materials is fixed inside the potential window of 33 mol kg^–1^ Na_0.45_K_0.55_FSA solution. KMnHCF showed two reversible redox peaks at approximately 0.7 and 1.2 V vs Ag/AgCl (Figure 4b). In addition, the CV curves at the first and second cycles almost overlapped, indicating high reversibility. NTP-C showed a couple of reversible redox peaks at approximately −0.7 V, and NVP-C showed a couple of reversible redox peaks at a lower potential of −1.1 V. These negative electrode materials also exhibited the second CV curves and almost overlapped with the initial one, demonstrating a highly reversible reaction. The mean potential difference between NTP-C and KMnHCF was approximately 1.7 V, and that between NVP-C and KMnHCF was approximately 2.1 V.

**Figure 5:**
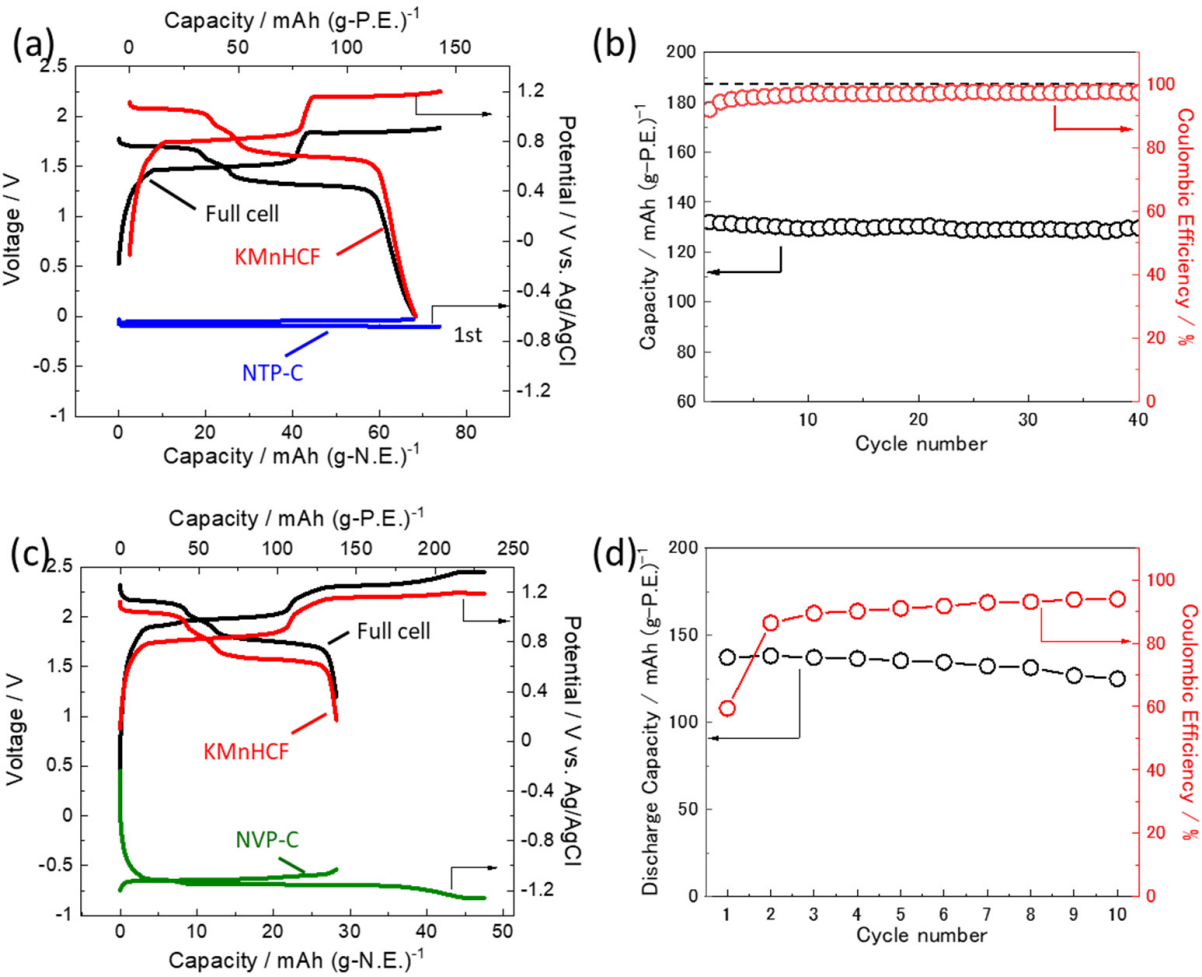
(a) Galvanostatic charge–discharge curves and (b) cycle performance and Coulombic efficiency of NTP-C|33 mol kg^–1^ Na_0.45_K_0.55_FSA/H_2_O|KMnHCF full cell at a current density of 156 mA (g-positive)^−1^. The mass loading ratio of negative and positive electrodes was fixed to *N*/*P* = 1.9:1.0. (c) Charge–discharge curves and (d) cycle performance and Coulombic efficiency of NVP-C|33 mol kg^–1^ Na_0.45_K_0.55_FSA/H_2_O|KMnHCF full cell. The mass loading ratio of negative and positive electrodes was fixed to *N*/*P* = 4.9:1.0.

As the redox reaction of the electrode materials was highly reversible, the aqueous full cells of NTP-C∥KMnHCF and NVP-C∥KMnHCF were fabricated and tested under galvanostatic charge and discharge conditions. Figure 5a displays the initial voltage profile of the NTP-C∥KMnHCF cell and the potential profile of NTP-C and KMnHCF electrodes in the full cell. Both NTP-C and KMnHCF electrodes showed highly reversible charge–discharge behavior, and the reversible capacities are 68 mAh (g-NTP-C)^−1^ and 132 mAh (g-KMnHCF)^−1^ (Figure 5a). The full cell exhibited two voltage plateaus at 1.7 and 1.3 V on discharge. Moreover, the full cell exhibited good cycle performance, maintaining 130 mAh (g-KMnHCF)^−1^ during >40 cycles, as shown in Figure 5b. The initial and subsequent Coulombic efficiencies are 90 and >97%, respectively. The increase in the Coulombic efficiency suggests that the partial irreversible capacity contributed to the formation of the SEI passivation layer on the negative electrode, which will be discussed later.

Figure 5c displays the initial voltage and potential profiles of the NVP-C∥KMnHCF full cell. Although the cell delivered a relatively large irreversible capacity at the initial cycle and consequent low Coulombic efficiency of ∼60%, NVP-C and KMnHCF electrodes exhibited reasonable reversible capacities of 29 mAh (g-NVP-C)^−1^ and 137 mAh (g-KMnHCF)^−1^. Most importantly, the full cell exhibited two voltage plateaus at 2.2 and 1.8 V in the discharge process. Thus, a 2 V-class aqueous Na/K dual-ion battery was demonstrated using 33 mol kg^–1^ Na_0.45_K_0.55_FSA. However, the reversible capacity slowly decreased to 125 mAh g^–1^ during 10 cycles, which would be due to partial electrolyte decomposition as the Coulombic efficiency remained around 94% in the subsequent cycles (Figure 5d). Further research on electrolyte and electrode materials is required to realize the longer cycles of 2 V-class aqueous Na/K-ion batteries.

As the electrolyte contains superconcentrated Na^+^ and K^+^ ions, both ions possibly insert as charge carriers during the redox reaction at the electrode materials. The negative electrode materials, NASICON-type NTP-C and NVP-C, are expected to have the preferential insertion of Na^+^ ions. Indeed, the charge–discharge profiles of the NTP-C and NVP-C electrodes in 33 mol kg^–1^ Na_0.45_K_0.55_FSA/H_2_O agreed well with those in nonaqueous Na cells but not in the K cells (Figure S3), suggesting preferential Na^+^-ion insertion. However, K^+^-ion insertion into NTP and NVP electrodes could occur in aqueous solutions as K^+^-ion insertion into NTP electrodes has been reported in a highly concentrated K solution.^7^ Thus, future studies should reveal the thermodynamic and kinetic effects on the insertion species in the dual-cation electrolytes. Highly reversible Na^+^ and K^+^ ion insertion into KMnHCF has been proven in nonaqueous Na^44^ and K^41^ cells, respectively. Thus, the charge–discharge mechanism of KMnHCF in the Na_0.45_K_0.55_FSA solution was examined by conducting ex situ XRD and EDX measurements.

**Figure 6:**
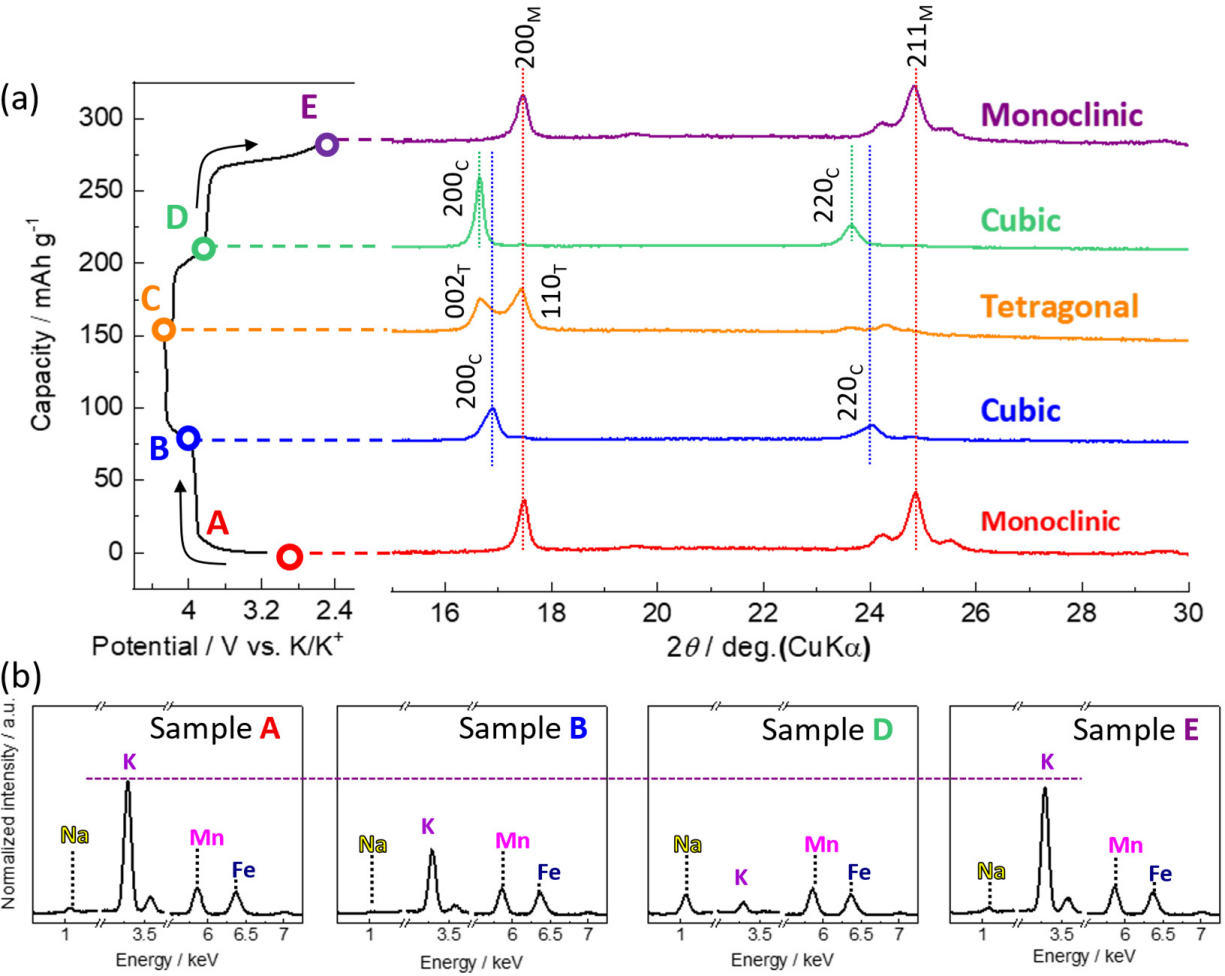
(a) Ex situ XRD patterns of KMnHCF electrode tested in 33 mol kg^–1^ Na_0.45_K_0.55_FSA electrolyte. (b) Ex situ SEM-EDX spectra of KMnHCF electrodes tested in 33 mol kg^–1^ Na_0.45_K_0.55_FSA electrolyte. Samples A–E correspond to points A–E in the charge–discharge curve of (a).

Figure 6 shows ex situ XRD patterns at different states of charge or discharge. The KMnHCF electrode soaked in the electrolyte (sample A in Figure 6) remained as a monoclinic structure identical to the pristine powder, indicating no apparent and spontaneous ionic exchange from the K^+^ ion to the Na^+^ ion. The KMnHCF electrode had a cubic structure at the end of the lower-voltage plateau (sample B), followed by phase transition into a tetragonal structure at the end of the charge process (1.2 V vs Ag/AgCl, sample C). This trend of structural evolution is identical to that of nonaqueous K cells.^41^ In the discharge process, KMnHCF reversibly transformed back into a cubic structure at the potential step (sample D). However, diffraction lines of 200_C_ and 220_C_ (in which C denotes the cubic phase) are located at a lower angle than the peaks of sample B. The different pattern suggests an asymmetric insertion/extraction mechanism. At the end of discharge (sample E), the electrode had a monoclinic structure identical to that in the initial state, showing reversible structural evolution to K_∼2_Mn[Fe(CN)_6_]. These results imply that the KMnHCF electrode undergoes asymmetric structural changes during the insertion/extraction process, though the initial monoclinic structure fully recovers after discharge.

**Figure 7:**
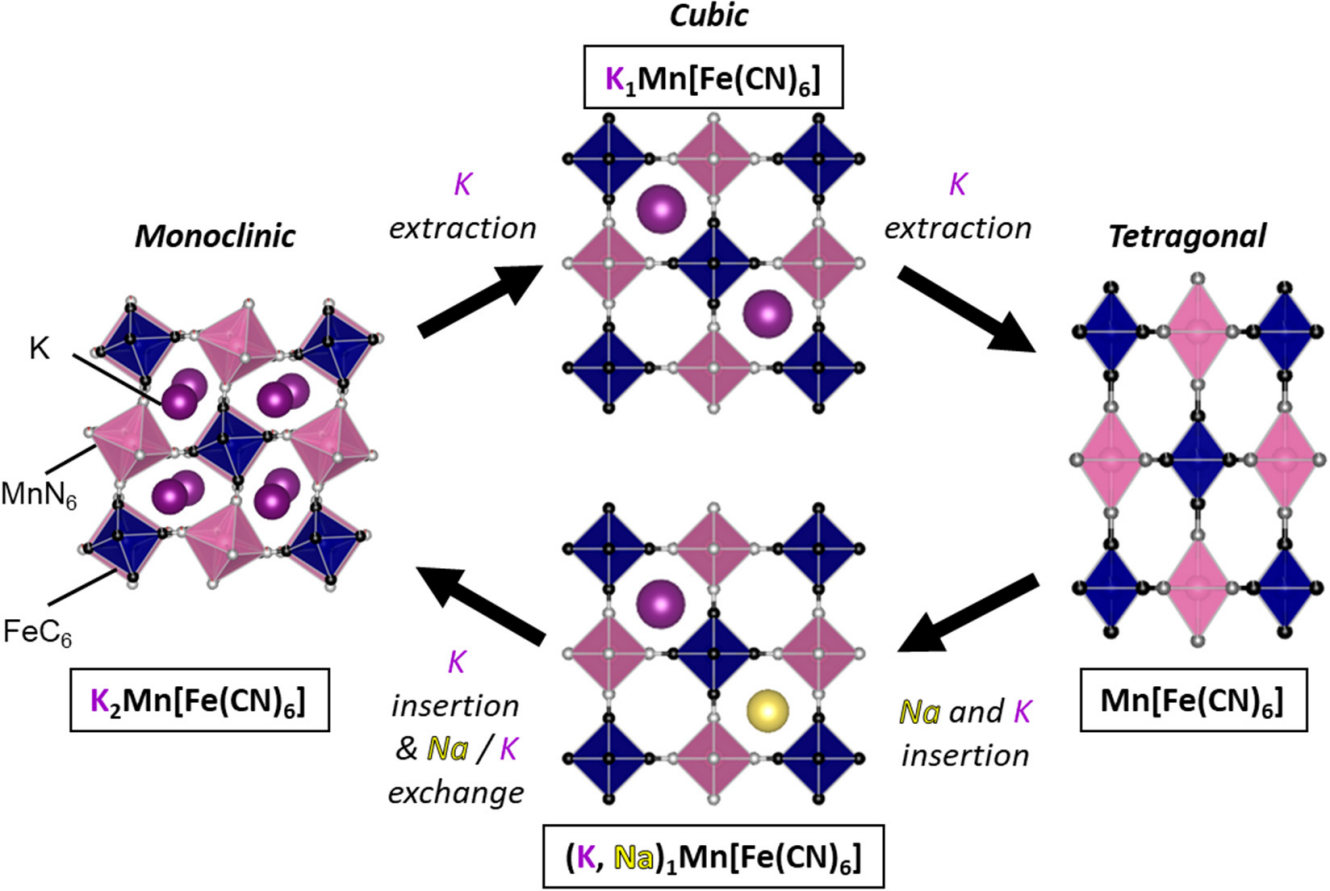
Crystal structures and phase transition of KMnHCF by electrochemical cation extraction and insertion in 33 mol kg^–1^ Na_0.45_K_0.55_FSA aqueous electrolyte.

To further understand the charge–discharge mechanism, EDX analysis was carried out for each point to probe the change in sodium and potassium contents (Figure 6b). All intensities were normalized by the peak intensity of Mn. The soaked electrode (sample A) exhibited the EDX peaks of K, Mn, and Fe, whereas the Na peak was negligible. This result confirms that the K/Na-ion exchange of KMnHCF hardly occurs in the electrolyte. In sample B, the K peak decreased, and the other peaks were unchanged by comparison to sample A, which revealed that K^+^ ions were extracted from KMnHCF. The ex situ XRD and EDX measurements show the evolution of the crystal structure from monoclinic to tetragonal through the cubic structure during K^+^-ion extraction, as shown in the schematic illustration (Figure 7). The Na peak intensity in the EDX spectrum of sample D was higher than that of K, which evidences partial Na^+^-ion insertion into KMnHCF at the higher potential discharge plateau. This result is consistent with the different XRD patterns of samples B and D as described above. However, the Na peak intensity was negligible after the end of the discharge (sample E), and that of K was the same as sample A. Therefore, K^+^ ions were mainly inserted for charge compensation during the redox reaction at the lower potential plateau, and the inserted Na^+^ ions were spontaneously exchanged to K^+^ ions (Figure 7). The complex charge–discharge mechanism can be explained as follows: K_2_Mn[Fe(CN)_6_] is thermodynamically more favorable than Na_2_Mn[Fe(CN)_6_].^43,45^ Compared with the A_2_Mn[Fe(CN)_6_] (A = Na or K) case, the thermodynamic stability difference between Na_1_Mn[Fe(CN)_6_] and K_1_Mn[Fe(CN)_6_] would be minor because the interaction between the alkali metal and framework is smaller in the lower alkali metal contents. Indeed, the Na_*x*_Mn[Fe(CN)_6_] electrode showed a redox potential accompanying Na^+^ insertion between Na_2_Mn[Fe(CN)_6_] and Na_1_Mn[Fe(CN)_6_] much lower than that of the K counterparts, whereas the Na_*x*_Mn[Fe(CN)_6_] electrode delivered a redox potential between Na_1_Mn[Fe(CN)_6_] and Na_0_Mn[Fe(CN)_6_] competitive with or even higher than that of the K counterparts (Figure S4). In the unique electrolyte of Na^+^ and K^+^ coexistence, therefore, both Na^+^ and K^+^ ions were inserted simultaneously at the higher voltage plateau, and K^+^ ions were mainly inserted at the lower discharge plateau, accompanied by Na^+^/K^+^-ion exchange. However, it should be noted that the insertion species may depend on kinetics such as charge/discharge rate. Future work should clarify the thermodynamic and kinetic effects on the insertion species into the positive electrode as well as the negative electrodes.

**Figure 8:**
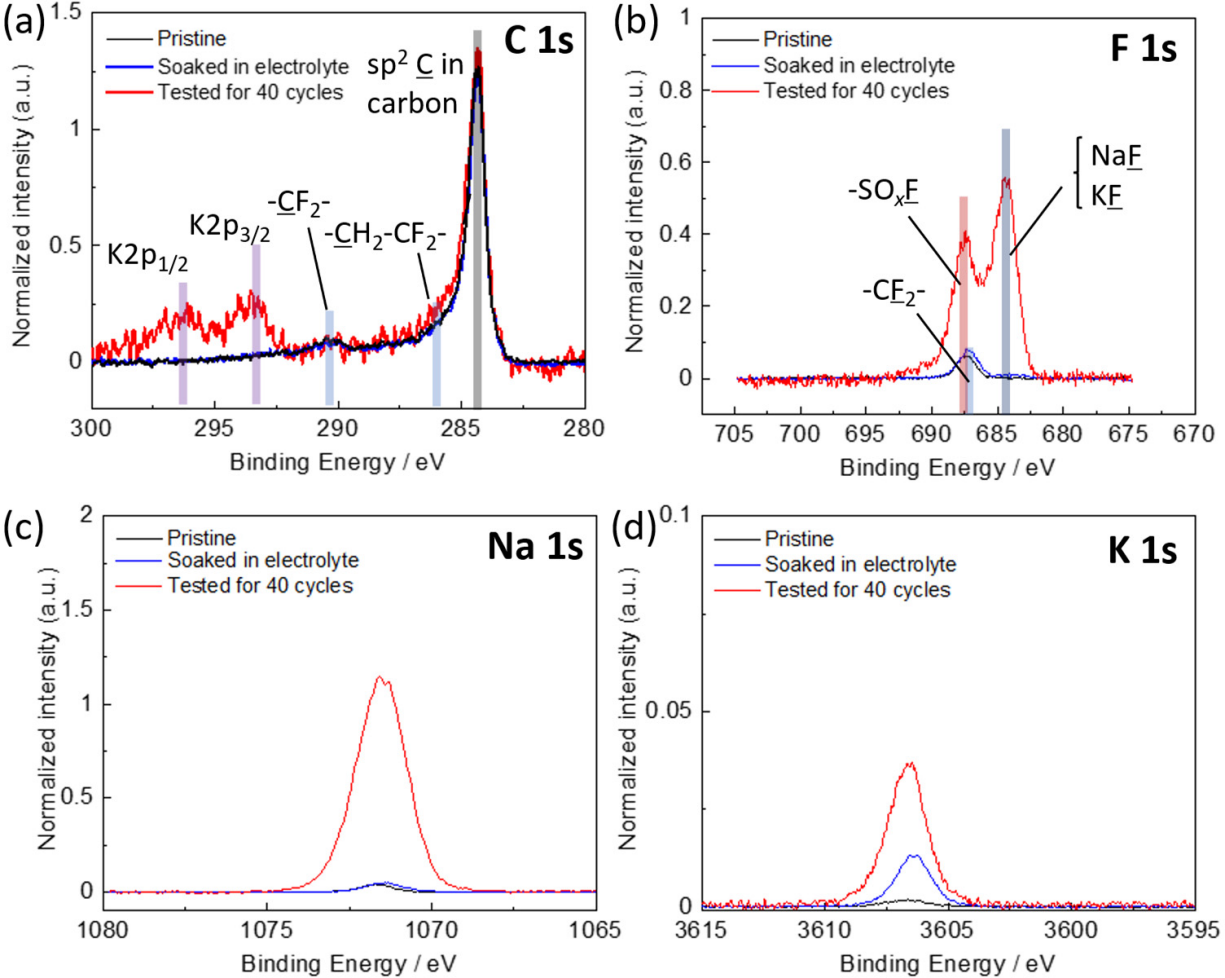
HAXPES spectra of NTP-C electrodes, pristine, soaked, and tested in 33 mol kg^–1^ Na_0.45_K_0.55_FSA electrolyte for 40 cycles: (a) C 1s and K 2p, (b) F 1s, (c) Na 1s, and (d) K 1s spectra.

Finally, HAXPES measurements were conducted to investigate surface chemistry on the NTP-C electrode surface. Figure 8 shows the HAXPES spectra of the electrodes: pristine, soaked in 33 mol kg^–1^ Na_0.45_K_0.55_FSA electrolyte, and tested for 40 cycles in the NTP-C∥KMnHCF cell. Intensities of all spectra were corrected by relative sensitivity factors^46^ and normalized by the integrated intensity of sp^2^ C peak at 284.3 eV to enable a semiquantitative analysis of the chemical species.^47^ In the C 1s/K 2p spectra of the pristine and soaked electrodes, peaks attributed to sp^2^ C, −CH_2_–CF_2_–, and CF_2_ (290.5 eV), which are derived from NTP-C/AB and PVDF, were observed (Figure 8a).^48^ The cycled electrode also showed no new peaks in the C 1s region, but peaks attributed to K 2p appeared in the range of 293–298 eV (Figure 8a), suggesting the deposition of the electrolyte decomposition products containing K species on the electrode. In the F 1s spectra, the peaks assigned to −SO_*x*_F (687.5 eV) and NaF/KF (684.0 eV) were observed for the cycled electrode, whereas only the peak attributed to PVDF-derived CF_2_ was observed in the pristine and soaked electrodes (Figure 8b). Moreover, a peak assigned to −SO_2_– was observed in the O 1s and S 1s spectra of the cycled electrode (Figure S5a,b). The new peaks, such as −SO_2_–, −SO_*x*_F, and NaF/KF, for the cycled electrode are attributed to the decomposition products of FSA^–^ anions.^49,50^ Moreover, clear peaks in Na 1s and K 1s spectra supported the formation of Na and K compounds like NaF/KF on the tested negative electrode surface (Figure 8c,d). These products would be a key component of the surface layer, including SEI, enabling charge and discharge with high Coulombic efficiency (Figure 5b), as reported previously in the case of nonaqueous and aqueous electrolytes.^3,50−52^ It is also worth noting that the relative atomic ratio of Na/sp^2^ C (2.2) was much higher than that of K/sp^2^ C (0.08), indicating that SEI contains more Na components such as NaF than K ones. The formation of a Na-rich SEI would be due to the lower solubility of NaF compared to that of KF as well as preferable Na insertion into NTP electrodes. Therefore, the addition of Na^+^ ions to K-ion electrolytes would be an effective strategy for stable SEI formation, which is supported by the cathodic stability of Na_1–*x*_K_*x*_FSA dual-ion electrolytes being higher than that of KFSA electrolyte, as shown in Figure 2b.

## Conclusions

Utilization of the eutectic composition of NaFSA–KFSA realized high concentration solutions of 35 mol kg^–1^ Na_0.55_K_0.45_FSA/H_2_O and 33 mol kg^–1^ Na_0.45_K_0.55_FSA/H_2_O. Both Na_1–*x*_K_*x*_FSA electrolytes show ionic conductivity of 20–25 mS cm^–1^, which is much higher than the reported superconcentrated Li solution. The 35 and 33 mol kg^–1^ Na_1–*x*_K_*x*_FSA solutions demonstrate the wider potential window of ∼3.5 V compared to that of the end-members. Based on the superior performance of the 33 mol kg^–1^ K_0.55_Na_0.45_FSA/H_2_O electrolyte, we demonstrated two different aqueous full cells of NTP-C∥KMnHCF and NVP-C∥KMnHCF configurations. The NTP∥KMnHCF cell shows reversible charge/discharge curves with mainly two discharge voltage plateaus located at 1.7 and 1.3 V and delivers high capacity retention. Although the NVP-C∥KMnHCF cell exhibits capacity degradation, it demonstrates 2 V-class operations. In these cells, Na^+^ ions were mainly inserted/extracted into/from the negative electrode, and both Na^+^ and K^+^ ions were inserted into the positive electrodes. The HAXPES measurements proved that the Na-rich SEI layer was formed on the negative electrode. The SEI would improve the cathodic stability of the electrolyte.

## Supporting Information Available

Experimental methods and results of semiquantitative analysis of fluoride ions, LSV curves of Pt and Ti working electrodes, charge–discharge curves of the NTP and NVP electrode in nonaqueous Na and K cells, CV curves of KMnHCF and NaMnHCF electrodes in NaFSA and KFSA aqueous electrolytes, O 1s and S 1s HAXPES spectra (PDF)
