# Supplementary material for: Superconcentrated NaFSA–KFSA Aqueous Electrolytes for 2 V-Class Dual-Ion Batteries
Source: ACS Appl Mater Interfaces. 2022 May 10;14(20):23507–17. doi: 10.1021/acsami.2c04289 (PMC9136840; doi:10.1021/acsami.2c04289)
Supplement: Supplementary file 1 — am2c04289_si_001.pdf [file am2c04289_si_001.pdf]

Supporting Information for

Superconcentrated NaFSA-KFSA Aqueous Electrolytes for 2 V-Class Dual-Ion Batteries

Tomooki Hosaka,[1,2] Ayumi Noda,[1] Kei Kubota,[1,2,†] Kento Chiguchi,[1] Yuki Matsuda,[3] Kazuhiko Ida,[3] Satoshi Yasuno,[4] and Shinichi Komaba*,[1,2]

[1] Department of Applied Chemistry, Tokyo University of Science, Shinjuku-ku, Tokyo 162-8601, Japan.

[2] Elements Strategy Initiative for Catalysts and Batteries (ESICB), Kyoto University, Nishikyo-ku, Kyoto 615-8245, Japan

[3] Technova Inc., Chiyoda-ku, Tokyo 100-0011, Japan

[4] Japan Synchrotron Radiation Research Institute (JASRI), SPring-8, 1-1-1 Kouto, Sayo-cho, Sayo-gun, Hyogo 679-5198, Japan

Present Address

[†]Center for Green Research on Energy and Environmental Materials (GREEN), National Institute for Materials Science (NIMS), 1-1 Namiki, Tsukuba, Ibaraki 305–0044, Japan

**Corresponding author: komaba@rs.tus.ac.jp*

Semi-quantitative analysis of fluoride ions

Detection of fluoride ions was conducted using Zr-EDTA complex and pyrocatechol violet.[1] Fluoride ion standard solutions were prepared by dissolving NaF (Kanto Chemical) in deionized water. $Zr(H_2O)_2EDTA$ solution was prepared by dissolving $ZrCl_4$ into 0.1 M Na_2H_2EDTA aqueous solution with Zr:EDTA = 1:1 (mol ratio) and stirring at 90 °C for several hours. Then, the solution was cooled to room temperature and diluting with deionized water to form 1 mM $Zr(H_2O)_2EDTA$ solution. A solution of 0.1 % pyrocatechol violet (Kishida Chemical) was diluted 2.59 times with deionized water. A buffer solution of 0.4 M acetic acid-sodium acetate solution was prepared by dissolving 0.4 mol acetic acid (FUJIFILM Wako Pure Chemical) and 0.4 mol sodium acetate trihydrate (FUJIFILM Wako Pure Chemical) in water to form 1 l solution. The analysis was conducted by mixing the standard or sample, Zr-EDTA, pyrocatechol violet, and buffer solutions in the volume ratio of 50:6:1:5. The absorbance spectra were recorded against a blank at a wavelength of 628 nm with UV-Vis spectrophotometer (SHIMAZU UV-3150PC). Before the solution preparation for the semi-quantitative analysis, the electrolyte was stored at 25 °C ± 3 °C for two weeks after preparation.

1. Balaji, T.; Matsunaga, H., Naked-eye detection of fluoride using Zr (IV)-EDTA complex and pyrocatechol violet. *Anal. Sci.* 2005, **21** (8), 973-977.

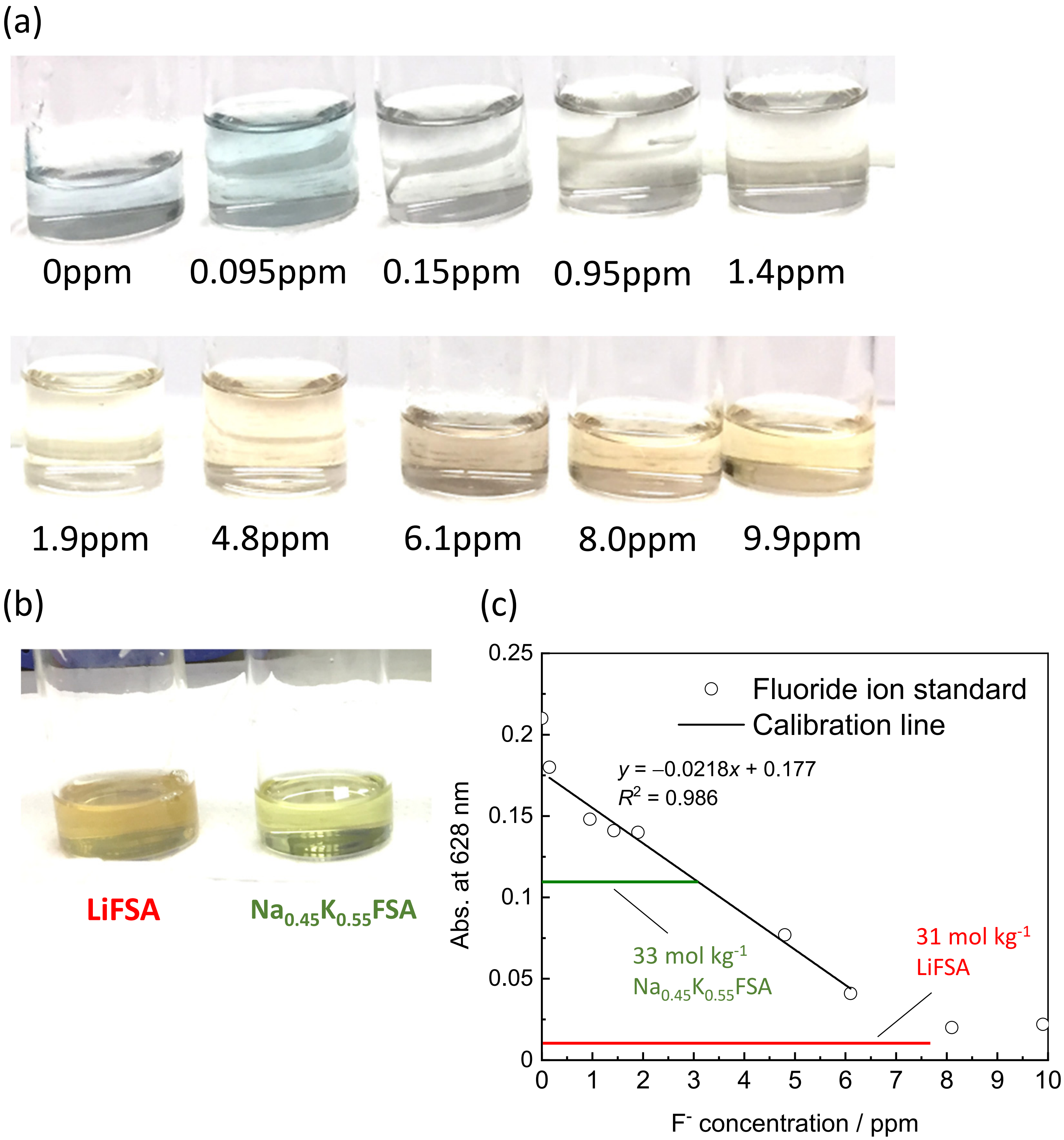

Figure S1. (a) Photograph of mixture solution of 0–10 ppm NaF, 1 mM Zr-EDTA, 1 mM pyrocatechol violet, acetic acid-sodium acetate buffer (pH 4.0). (b) Photograph of mixture solution of 31 mol kg^{-1} LiFSA or 33 mol kg^{-1} $Na_{0.45}K_{0.55}FSA$, 1 mM Zr-EDTA, 1 mM pyrocatechol violet, acetic acid-sodium acetate buffer. The volume ratio of NaF or sample electrolyte : Zr-EDTA : pyrocatechol violet : buffer solutions were 50:6:1:5. (c) Absorbance at a wavelength of 628 nm of the solutions. The absorbance of standard solutions showed almost linear relationship with the concentrations between 0.0095 ppm and 6.1 ppm. The absorbance indicated the F^- concentration was 3–4 ppm in 33 mol kg^{-1} $Na_{0.45}K_{0.55}FSA$. The absorbance of 31m LiFSA was too low and the F^- concentration exceeds the upper limit of the calibratable concentration (6 ppm).

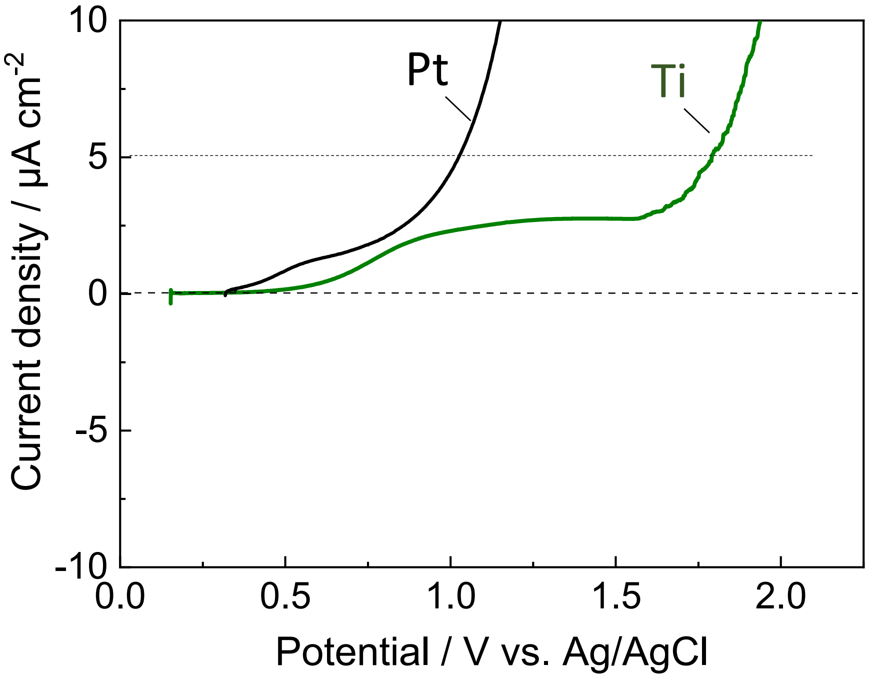

Figure S2. LSV curves in 35 mol kg^{-1} $Na_{0.55}K_{0.45}FSA/H_2O$ solutions with different working electrodes at a scan rate of 0.5 mV s^{-1}. The working electrodes were Pt (black curve) and Ti (green curve) foils.

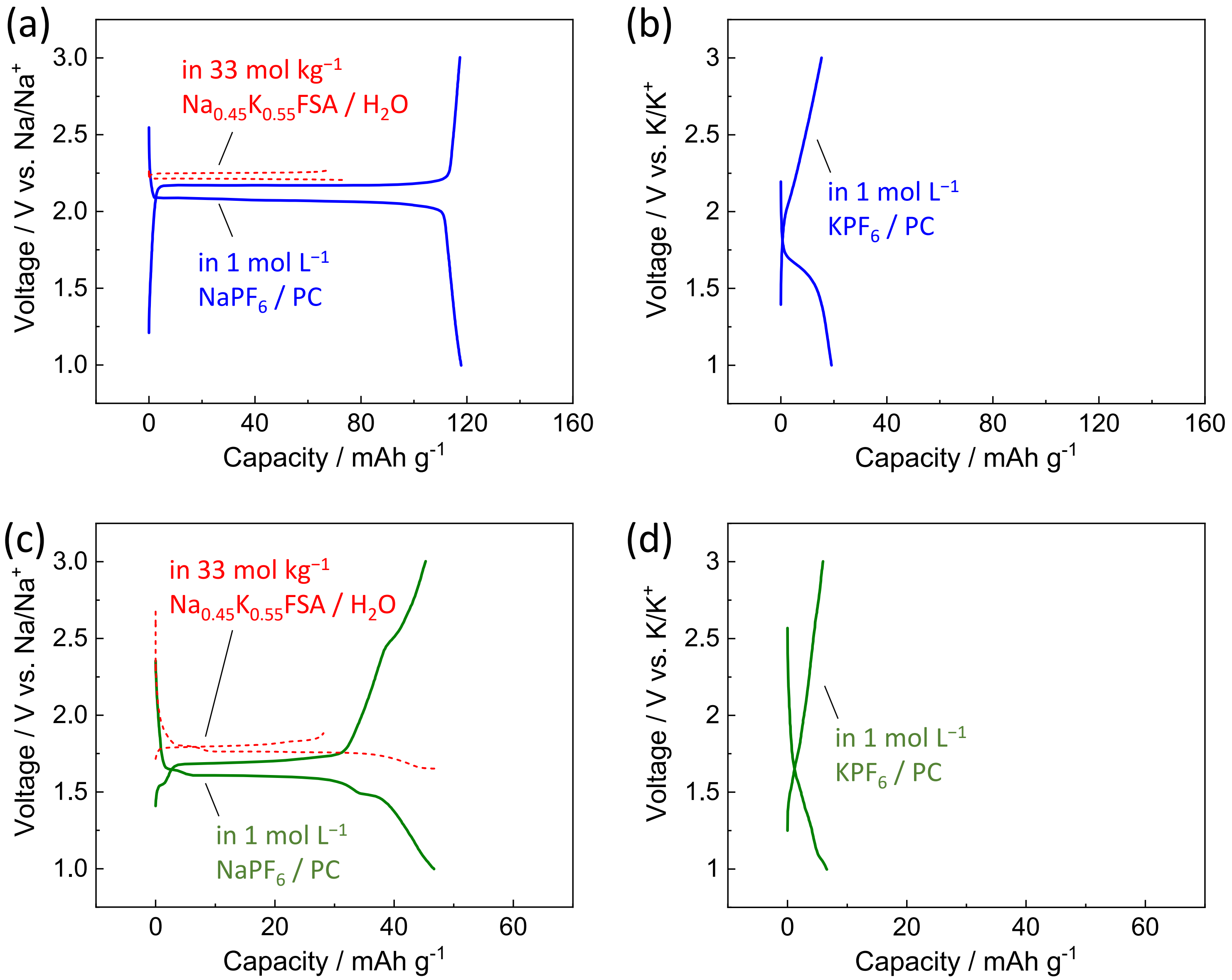

Figure S3. Charge-discharge curves of NTP-C electrode in (a) 1 M $NaPF_6$/PC and (b) 1 M KPF_6/PC electrolytes. Charge-discharge curves of NVP-C electrode in (c) 1 M $NaPF_6$/PC and (d) 1 M KPF_6/PC electrolytes. The charge-discharge curves of NTP-C and NVP-C in 33 mol kg^{-1} $Na_{0.45}K_{0.55}FSA$ are also shown in (a) and (b) for comparison.

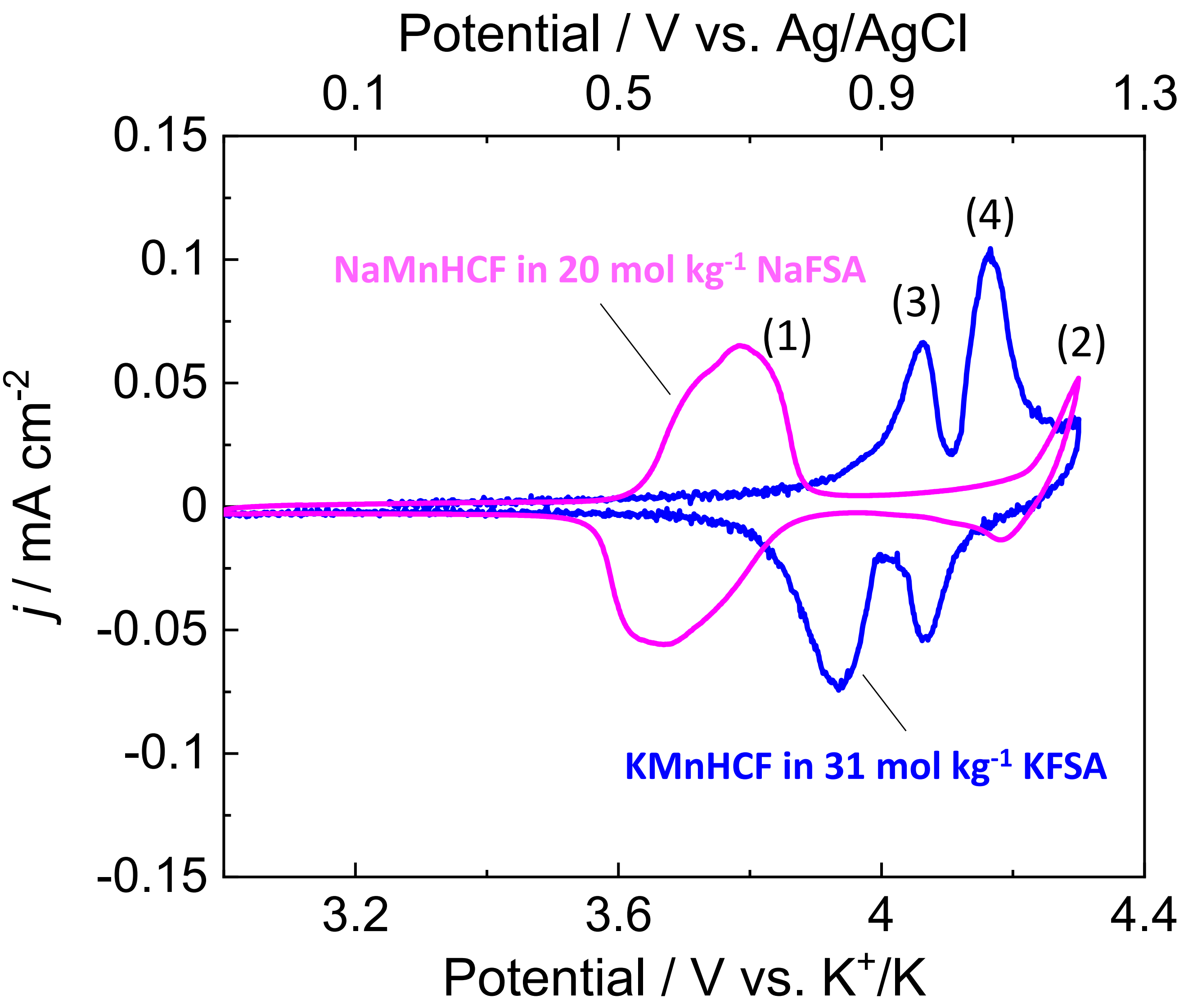

Figure S4. Cyclic voltammograms of KMnHCF electrode in 31 mol kg^{-1} KFSA solution and NaMnHCF in 20 mol kg^{-1} NaFSA solution at a scan rate of 0.1 mV s^{-1}. The redox couples labeled as (1)–(4) correspond to the reactions in the following equations (1)– (4), respectively.

$$Na_2Mn[Fe(CN)_6] \rightleftarrows Na_1Mn[Fe(CN)_6] + Na^+ + e^- \quad (1)$$

$$Na_1Mn[Fe(CN)_6] \rightleftarrows Mn[Fe(CN)_6] + Na^+ + e^- \quad (2)$$

$$K_2Mn[Fe(CN)_6] \rightleftarrows K_1Mn[Fe(CN)_6] + K^+ + e^- \quad (3)$$

$$K_1Mn[Fe(CN)_6] \rightleftarrows Mn[Fe(CN)_6] + K^+ + e^- \quad (4)$$

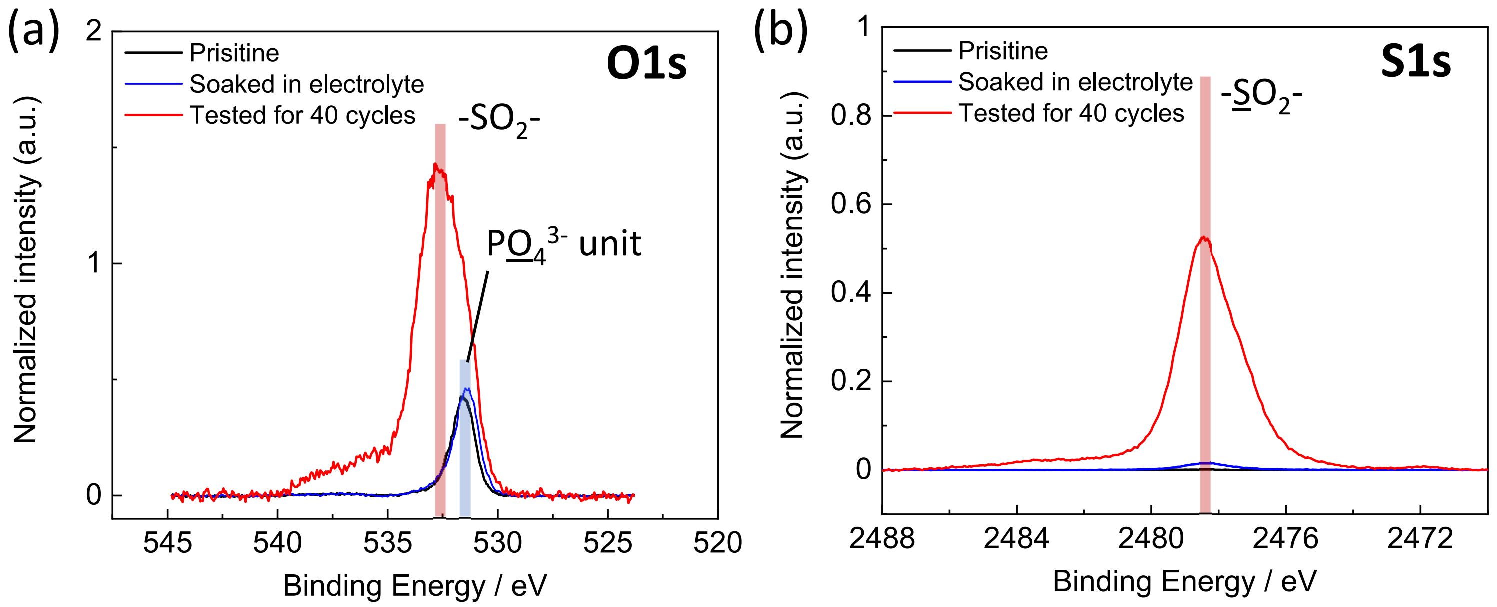

Figure S5. HAXPES spectra of NTP-C electrodes, pristine, soaked, and tested in 33 mol kg^{-1} $Na_{0.45}K_{0.55}$FSA electrolyte for 40 cycles: (a) O 1s and (b) S 1s spectra.
